# Effect of mTORC Agonism via MHY1485 with and without Rapamycin on C2C12 Myotube Metabolism

**DOI:** 10.3390/ijms25136819

**Published:** 2024-06-21

**Authors:** Norah E. Cook, Macey R. McGovern, Toheed Zaman, Pamela M. Lundin, Roger A. Vaughan

**Affiliations:** 1 Department of Health and Human Performance, High Point University, High Point, NC 27262-3598, USA; ncook@highpoint.edu (N.E.C.); mmcgove4@highpoint.edu (M.R.M.); 2 Department of Chemistry, High Point University, High Point, NC 27262-3598, USA; tzaman@highpoint.edu (T.Z.); plundin@highpoint.edu (P.M.L.)

**Keywords:** leucine, isoleucine, valine, skeletal muscle, branched-chain amino acids, mitochondrial function

## Abstract

The mechanistic target of rapamycin complex (mTORC) regulates protein synthesis and can be activated by branched-chain amino acids (BCAAs). mTORC has also been implicated in the regulation of mitochondrial metabolism and BCAA catabolism. Some speculate that mTORC overactivation by BCAAs may contribute to insulin resistance. The present experiments assessed the effect of mTORC activation on myotube metabolism and insulin sensitivity using the mTORC agonist MHY1485, which does not share structural similarities with BCAAs. Methods: C2C12 myotubes were treated with MHY1485 or DMSO control both with and without rapamycin. Gene expression was assessed using qRT-PCR and insulin sensitivity and protein expression by western blot. Glycolytic and mitochondrial metabolism were measured by extracellular acidification rate and oxygen consumption. Mitochondrial and lipid content were analyzed by fluorescent staining. Liquid chromatography-mass spectrometry was used to assess extracellular BCAAs. Results: Rapamycin reduced p-mTORC expression, mitochondrial content, and mitochondrial function. Surprisingly, MHY1485 did not alter p-mTORC expression or cell metabolism. Neither treatment altered indicators of BCAA metabolism or extracellular BCAA content. Conclusion: Collectively, inhibition of mTORC via rapamycin reduces myotube metabolism and mitochondrial content but not BCAA metabolism. The lack of p-mTORC activation by MHY1485 is a limitation of these experiments and warrants additional investigation.

## 1. Introduction

The mechanistic target of rapamycin complexes (mTORC1 and TORC2) are heavily researched protein complexes for their roles in cell metabolism which have importance for disease and human performance. mTORC1 activation has been routinely linked with increased protein synthesis, which has implications for skeletal muscle hypertrophy. Mechanistically, mTORC1 is sensitive to changes in nutrients and can be activated by several stimuli, including nutrient abondance and the accompanying hormonal milieu. For example, the branched-chain amino acid (BCAA) leucine appears to activate mTORC1 in a number of ways, including acetyl-CoA synthesis [1], by activating the GAP Activity Towards Rags (GATOR) complex (a positive regulator of mTOR); by inhibiting SESTRIN2 [2] or Secretion Associated Ras Related GTPase 1B (SAR1B) [3], which negatively regulates GATOR; or by suppressing AMP-activated protein kinase (AMPK) [4]. Conversely, when energy abundance is lower, AMPK is activated by increased ratios of ADP:ATP, such as those associated with reduced energy intake and/or exercise [5,6,7], which is associated with reduced mTORC1 activation.

Interestingly, activation of mTORC1 has also been linked with increased mitochondrial function. For example, activation of mTORC1 via tuberous sclerosis 2 (TSC2) silencing increased mitochondrial biogenesis and function [8]. Specifically, TSC2^−/−^ increased the expression of peroxisome proliferator-activated receptor gamma coactivator 1-alpha (PGC-1α) [8], which is a primary regulator of mitochondrial biogenesis, by controlling the expression of nuclear respiratory factors (NRF1/2) [9,10,11] and mitochondrial transcription factor A (TFAM) [10,12,13]. The report by Cunningham et al. elegantly showcased that activation of mTORC1 increased expression of several mitochondrial genes as well as mitochondrial DNA [8]. Cunningham et al. also showed that the effect could be depressed with the addition of rapamycin (demonstrating mTORC dependence) [8]. Others have also observed a negative effect of mTORC inhibition via rapamycin on mitochondrial function [14]. Given that PGC-1α activity is regulated in part by AMPK phosphorylation [15], which is typically thought to oppose mTORC activity [16], it is surprising that mTORC activation appears to cooperate with AMPK to coordinate increased mitochondrial content and function. Furthermore, PGC-1α knockout mice display reduced activation of downstream targets of mTORC1 following leucine ingestion (which activated protein synthesis in wild type littermates [17]), which suggests a coordinated process by which anabolic and catabolic processes support one another (which has been discussed previously [18]).

Though required in the human diet, a consistent relationship between elevated circulating BCAA concentrations and severity of insulin resistance has been observed (reviewed elsewhere [19,20,21,22,23,24,25,26,27,28]). The relationship between elevated BCAA and insulin resistance has been known for some time [29], yet the exact cause of the relationship remains somewhat controversial, as multiple mechanisms may contribute. One hypothesis is that chronic BCAA-mediated stimulation of mTORC1 leads to continuous insulin receptor substrate (IRS) activation, thereby reducing insulin sensitivity [22]. However, other observations have implicated the accumulation of acyl-carnitine and mitochondrial dysfunction [23,30], the production of 3-hydroxyisobutyrate-mediated lipid accumulation [31,32,33,34], and the inhibition of mitochondrial function as other potential mechanisms by which BCAAs may worsen insulin resistance [22,23]. This has led many to seek small molecule therapeutics that promote BCAA metabolism, such as BT2 or 4-PBA [35,36,37,38,39,40,41]. 

Interestingly, mTORC1 has been shown to govern regulators of BCAA catabolism. The most direct measurement of the ability of mTORC1 to regulate BCAA metabolism was demonstrated by Zhen et al., who showed that leucine administration increased both downstream targets of mTORC activation and branched-chain α-ketoacid dehydrogenase complex (BCKDH) activity [42], the rate-limiting enzyme in BCAA catabolism. The report further demonstrated that the enhanced activity of BCKDH could be abolished by rapamycin, suggesting the direct involvement of mTORC [42]. One important consideration, however, is that mTORC activation was achieved via leucine administration. Thus, while these observations suggest that mTORC positively activates BCAA metabolism, it is unclear if the activation of BCKDH by leucine is a result of it also being metabolized by BCKDH (following transamination). Moreover, leucine and other activators of mTORC1 increase mitochondrial content and function (reviewed elsewhere [43]). Therefore, it is worth exploring if the effect of mTORC activation on BCAA catabolic capacity occurs independent of additional BCAAs (namely leucine). It is also unclear if reduced BCKDH activity by RAPA is a byproduct of its negative effect on mitochondrial metabolism (versus direct regulation of BCKDH). Therefore, the purpose of these experiments was to assess the effect of the mTORC1 agonist on myotube metabolism and insulin sensitivity using an mTORC agonist that does not share chemical resemblance to any BCAA. Specifically, this report will assess the effects of MHY1485, which has been shown to activate mTORC [44,45], both with and without mTORC inhibition via rapamycin treatment. 

## 2. Results

### 2.1. Effect of MHY1485 on Myotube Physiology, Viability, and Differentiation

**Figure 1:**
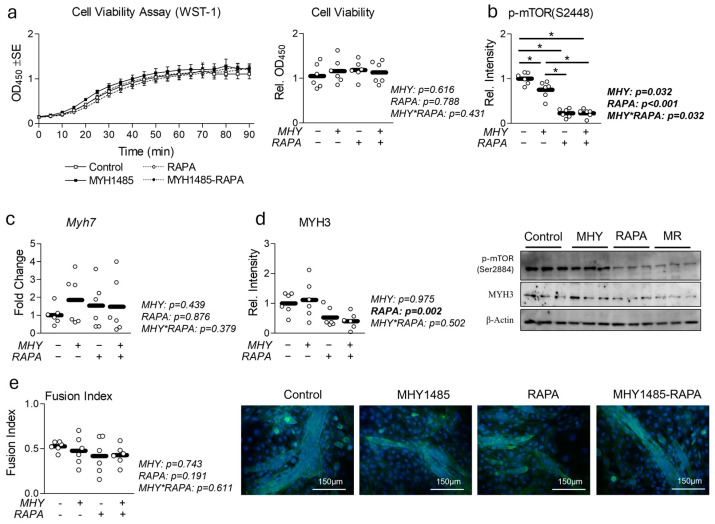
Effect of MHY1485 on myotube viability and differentiation. (**a**) Effect of MHY1485 (MHY) at 10 μM both with and without rapamycin (RAPA) at 100 nM (final concentration of DMSO at 0.2% for all samples) for 24 h on cell viability presented as time trial (left) or end point (right). (**b**) Effect of MHY with or without RAPA for 24 h on p-MTORC expression. (**c**) Effect of MHY with or without RAPA for 24 h on myosin heavy chain 7 (Myh7) mRNA expression. (**d**) Effect of MHY with or without RAPA for 24 h on MYH3 protein expression. (**e**) Effect of MHY with or without RAPA for 24 h on myotube fusion index. NOTES: Data were analyzed using two-way ANOVA followed by one-way ANOVA with Bonferroni’s correction for multiple comparisons to assess differences in each outcome. * Indicates a significant difference between groups upon pair-wise comparisons. Data were generated from three replicates per group across two independent experiments with n = 6 for the final analysis. Target gene expression was normalized to TATA-binding protein (Tbp), which did not differ between groups (Figure S1). Protein expression was normalized to β-Actin, which did not differ between groups (Figure S2). Myotube fusion was quantified by two blinded members of the research team. No differences were observed between any groups. Images in (**e**) of representative individual myotubes were taken using the 20× objective.

To begin, we assessed the effects of MHY1485 (MHY) on myotube viability and differentiation with and without the presence of rapamycin (RAPA) (Figure 1). Cell viability remained unaltered by each treatment (Figure 1a), however, a reduction in p-mTORC (Figure 1b) and myosin heavy chain 3 (MYH3) protein expression (Figure 1d) was observed following RAPA treatment. Surprisingly, we observed no significant effect of MHY1485 on p-mTORC or MYH3 expression (Figure 1b,d). Finally, we assessed the effect of each treatment on myotube formation and found no significant difference in myotube fusion index across any treatment condition (Figure 1e).

### 2.2. Effect of MHY1485 on Myotube Mitochondrial Metabolism, Content, and Biogenesis

**Figure 2:**
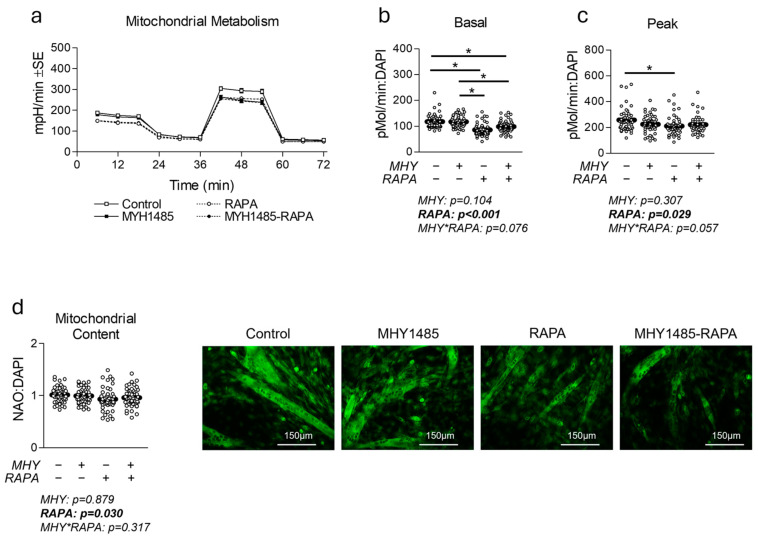
Effect of MHY1485 on mitochondrial content and function. (**a**) Time course of the effect of MHY1485 (MHY) at 10 μM both with and without rapamycin (RAPA) at 100 nM (final concentration of DMSO at 0.2% for all samples) for 24 h on mitochondrial function. (**b**,**c**) Effect of treatment as described in (**a**) on basal (**b**) and peak (**c**) mitochondrial metabolism following normalization to cell nuclei content (presented in Figure S3). (**d**) Mitochondrial content of cells described in (**a**) indicated by NAO staining following normalization to cell nuclei content (presented in Figure S3). NOTES: Data were analyzed using two-way ANOVA followed by one-way ANOVA with Bonferroni’s correction for multiple comparisons to assess differences in each outcome. * Indicates a significant difference between groups upon pair-wise comparisons. States of mitochondrial metabolism were calculated by subtracting non-mitochondrial respiration from basal or FCCP-induced peak oxygen consumption. Metabolic measurements were performed using n = 23 individual replicates per treatment condition and were repeated across two independent experiments with n = 46 per group in the final analyses. No wells responded with negative raw values. Mitochondrial staining was performed using n = 23 individual replicates per treatment condition and were repeated across two independent experiments with n = 46 per group in the final analyses using the average of three measurements per experiment less background. Images in (**d**) of representative individual myotubes were taken using the 20× objective.

**Figure 3:**
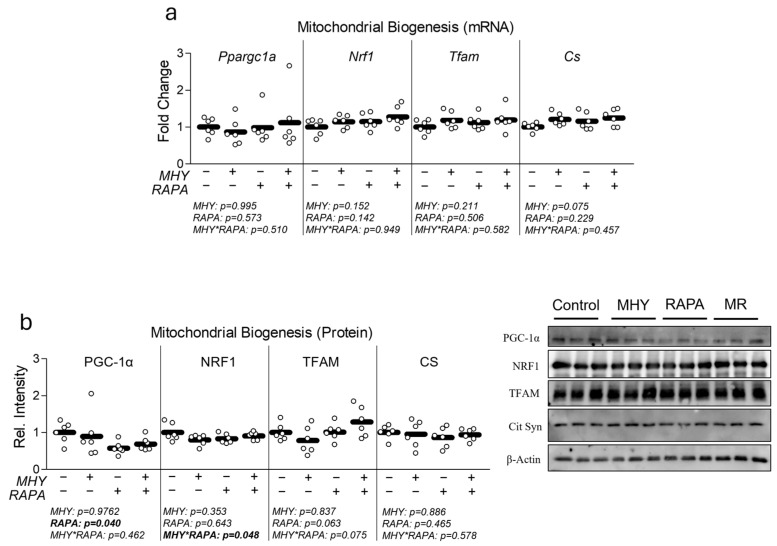
Effect of MHY1485 on mitochondrial biogenic signaling. (**a**) Effect of MHY1485 (MHY) at 10 μM both with and without rapamycin (RAPA) at 100 nM (final concentration of DMSO at 0.2% for all samples) for 24 h on mRNA expression of mitochondrial biogenesis including peroxisome proliferator-activated receptor-gamma coactivator-1alpha (Ppargc1a), nuclear respiratory factor 1 (Nrf1), mitochondrial transcription factor A (Tfam), and citrate synthase (Cs). (**b**) Effect of MHY with or without RAPA for 24 h on protein expression of peroxisome proliferator-activated receptor-gamma coactivator-1alpha (PGC-1α), nuclear respiratory factor 1 (NRF1), mitochondrial transcription factor A (TFAM), and citrate synthase (CS). NOTES: Data were analyzed using two-way ANOVA followed by one-way ANOVA with Bonferroni’s correction for multiple comparisons to assess differences in each outcome. Data were generated from three replicates per group across two independent experiments with n = 6 for the final analysis. Target gene expression was normalized to TATA-binding protein (Tbp), which did not differ between groups (Figure S1). Protein expression was normalized to β-Actin, which did not differ between groups (Figure S2).

Because mTOR has been shown to affect mitochondrial metabolism, we next measured the effect of MHY1485 on mitochondrial metabolism, content, and biogenesis (Figure 2). RAPA caused a significant decrease in both the basal and peak mitochondrial metabolism (Figure 2b,c), as well as significant reduction in mitochondrial content (Figure 2d). Meanwhile, MHY1485 treatment had no significant effect on mitochondrial function or content (Figure 2). To further explore the effect of each treatment on mitochondrial function and content, we measured indicators of mitochondrial biogenic signaling (Figure 3). At the mRNA level, no significant effects were observed for any treatment (Figure 3a); however, at the protein level, PGC-1α was depressed in RAPA-treated cells (Figure 3b).

### 2.3. Effect of MHY1485 on Myotube Glycolytic Metabolism and Insulin Sensitivity

**Figure 4:**
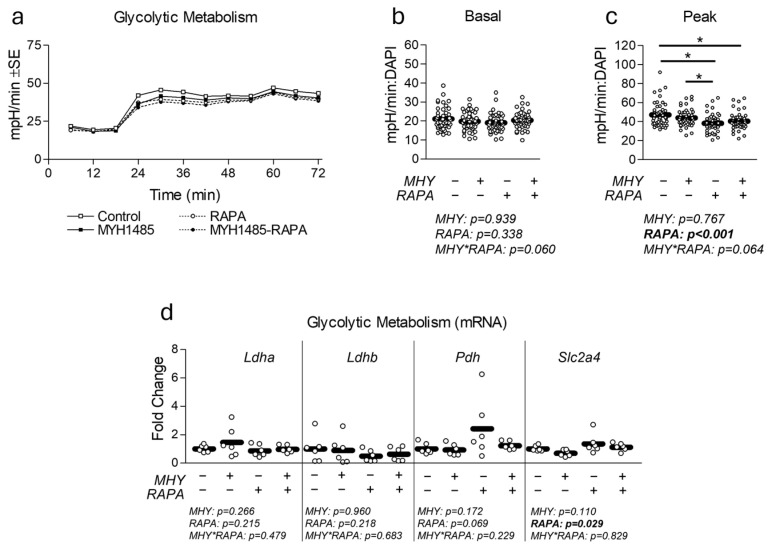
Effect of MHY1485 on glycolytic metabolism. (**a**) Time course of the effect of MHY1485 (MHY) at 10 μM both with and without rapamycin (RAPA) at 100 nM (final concentration of DMSO at 0.2% for all samples) for 24 h on glycolytic metabolism. (**b**,**c**) Effect of treatment as described in (**a**) on basal (**b**) and peak (**c**) glycolytic metabolism following normalization to cell nuclei content (presented in Figure S3). (**d**) Effect of treatment as described in (**a**) on mRNA expression of glycolytic metabolism including lactate dehydrogenase a (Ldha), lactate dehydrogenase b (Ldhb), pyruvate dehydrogenase (Pdh), and glucose transporter 4 (Slc2a4/Glut4). NOTES: Data were analyzed using two-way ANOVA followed by one-way ANOVA with Bonferroni’s correction for multiple comparisons to assess differences in each outcome. * Indicates a significant difference between groups upon pair-wise comparisons. Metabolic measurements were performed using n = 23 individual replicates per treatment condition and were repeated across two independent experiments with n = 46 per group in the final analyses. No wells responded with negative raw values. Gene expression data were generated from three replicates per group across two independent experiments with n = 6 for the final analysis. Target gene expression was normalized to TATA-binding protein (Tbp), which did not differ between groups (Figure S1).

**Figure 5:**
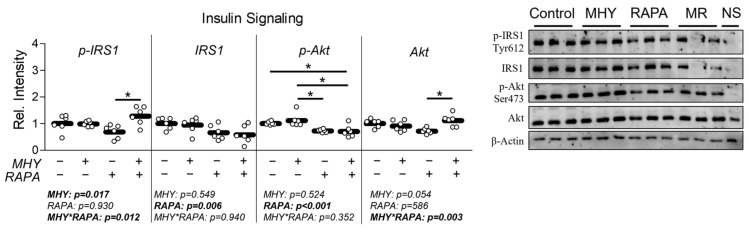
Effect of MHY1485 on insulin sensitivity. The effect of MHY1485 (MHY) at 10 μM both with and without rapamycin (RAPA) at 100 nM (final concentration of DMSO at 0.2% for all samples) for 24 h followed by insulin stimulation at 100 nM for 30 min on pIRS1 (Tyr612) expression and pAkt (Ser473) expression. NOTES: Data were analyzed using two-way ANOVA followed by one-way ANOVA with Bonferroni’s correction for multiple comparisons to assess differences in each outcome. * Indicates a significant difference between groups upon pair-wise comparisons. Data were generated from three replicates per group across two independent experiments with n = 6 for the final analysis. Protein expression of phosphorylated protein targets was normalized to either total IRS1 or total Akt, and total IRS1 and total Akt expression were normalized to β-Actin. Non-stimulated (NS) negative control is also displayed on the far-right lane of each blot.

Because mTOR activity is implicated in the regulation of insulin sensitivity, we next measured the effect of MHY1485 on glycolytic metabolism (Figure 4) and insulin sensitivity (Figure 5). Consistent with changes in mitochondrial function, RAPA decreased peak glycolytic function; however, this occurred without significant change in the gene expression of targets associated with glycolytic metabolism (Figure 4c and Figure 4d, respectively). Surprisingly, however, RAPA treatment resulted in a significant increase in Glut4 (Slc2a4) mRNA expression (Figure 4d). Similar to mitochondrial function experiments, MHY1485 treatment had no significant effect on glycolytic metabolism (Figure 4a–c) or glycolytic mRNA expression (Figure 4d). To assess the effect of mTORC manipulation on insulin sensitivity, we measured the activation of pIRS-1 and pAkt expression following 30 min insulin stimulation. pIRS-1 activation showed a significant effect of MHY1485 as well as a significant interaction effect (Figure 5). Conversely, pAkt expression was significantly depressed by RAPA treatment but unaltered by MHY1485 (Figure 5). 

### 2.4. Effect of MHY1485 on Myotube BCAA Catabolism

**Figure 6:**
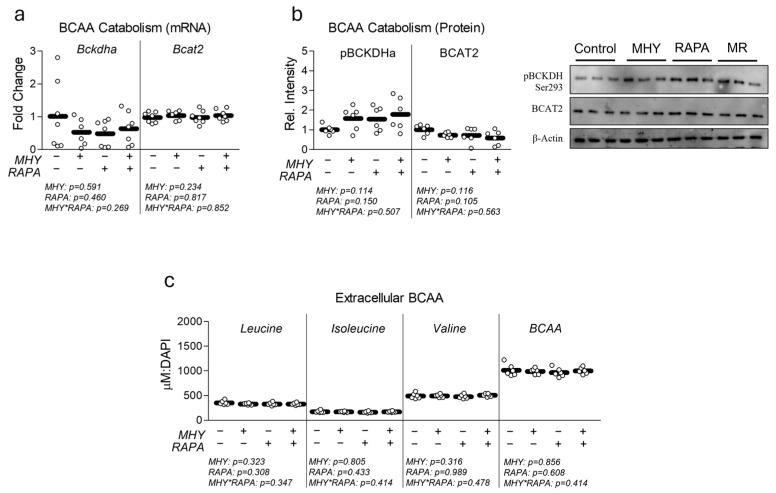
Effect of MHY1485 on BCAA catabolism. (**a**) Effect of MHY1485 (MHY) at 10 μM both with and without rapamycin (RAPA) at 100 nM (final concentration of DMSO at 0.2% for all samples) for 24 h on mRNA expression of branched-chain alpha-keto acid dehydrogenase (Bckdha) and branched-chain amino transaminase 2 (Bcat2). (**b**) Effect of MHY with or without RAPA for 24 h on protein expression of phospho-branched-chain alpha-keto acid dehydrogenase (pBCKDHa) and branched-chain amino transaminase 2 (BCAT2). (**c**) Effect of MHY with and without RAPA as described in (**a**) on extracellular BCAA content following normalization to relative nuclei content (Figure S4). NOTES: Data were analyzed using two-way ANOVA followed by one-way ANOVA with Bonferroni’s correction for multiple comparisons to assess differences in each outcome. Data were generated from three replicates per group across two independent experiments with n = 6 for the final analysis. Target gene expression was normalized to TATA-binding protein (Tbp), which did not differ between groups (Figure S1). Protein expression was normalized to β-Actin, which did not differ between groups (Figure S2). Extracellular BCAAs were normalized to nuclei content (Figure S4).

Finally, because of the relationship between BCAAs and mTOR, we measured the effect of MHY1485 on myotube BCAA catabolism (Figure 6). To our surprise, neither MHY1485 nor RAPA had a significant effect on the mRNA (Figure 6a) or protein (Figure 6b) expression of primary BCAA catabolic enzymes. In line with these observations, neither treatment altered the abundance of extracellular BCAAs (Figure 6c), suggesting that BCAA metabolism did not differ between the groups.

## 3. Discussion

mTORC1 is an important protein complex that coordinates appreciable aspects of protein synthesis in response to changes in nutrients and related hormonal signaling. BCAAs such as leucine are examples of nutrients that activate mTORC1 via multiple mechanisms. Activation of mTORC1 has also been implicated in the activation of IRS-1 leading to a desensitization to insulin signaling. Along with anabolic signaling associated with insulin signaling and protein synthesis, activation and/or inhibition of mTORC1 has also been linked with altered mitochondrial function [8,14]. During insulin resistance, the reduced sensitivity of target tissues to insulin, the disrupted mitochondrial function, and the accumulation of BCAA suggest a potential relationship between the variables. In this report, we assessed the effect of activation and inhibition of the mTORC1 complex using either the mTORC1 activator MHY1485 and/or the established mTORC1 inhibitor, rapamycin. While rapamycin elicited the expected response of reduced mTORC activation, MHY1485 did not stimulate mTORC activation, which was a surprising finding. Similar peculiar findings have been observed with another reported mTORC1 stimulator, 3-Benzyl-5-((2-nitrophenoxy)methyl)-dihydrofuran-2(3H)-one (3BDO) [46]. Specifically, L6 myotubes treated with and without 3BDO showed reduced p-mTORC1 expression versus control cells under conditions free of insulin stimulation [46]. Thus, while it was anticipated that MHY1485 would activate mTORC1, it is conceivable that the conditions were not optimal to elicit this effect. 

Secondly, we observed a consistent reduction in both mitochondrial metabolism and content following mTORC inhibition via rapamycin that is consistent with previous observations in similar models [8,14]. Surprisingly, reduced mitochondrial metabolism and content occurred without detectable alterations in regulators of mitochondrial biogenesis, except for PGC-1α, which exhibited a small but significant reduction in rapamycin-treated cells (indicated by the significant main effect). Similarly, rapamycin-treated cells displayed reduced glycolytic metabolism, which also occurred without significant expressional changes in related mRNA transcripts. Additionally, seemingly divergent alterations in insulin signaling (pIRS-1 and pAkt activation) were observed, suggesting a disruption in the insulin signaling axis during the different conditions. This could also be in part resulting from the disparity in activation of Akt by mTORC1 and mTORC2 and the potential lack of specificity of rapamycin in the inhibition of both mTOR complexes [14]. That said, pAkt expression was depressed in rapamycin-treated cells following insulin stimulation, suggesting reduced insulin sensitivity, which is in line with other observations [14]. 

Along with mitochondrial metabolism and insulin sensitivity, mTORC1 has been shown to govern regulators of BCAA catabolism [42]. However, one important consideration in these experiments is that mTORC activation was achieved via leucine treatment. Given that leucine is metabolized predominantly in the mitochondria and has previously been associated with altered mitochondrial function, we assessed the effect of mTORC1 activation on indicators of BCAA metabolism using MHY1485, which does not resemble any BCAA in structure [44,45]. Consistent with our other metabolic findings, MHY1485 had no effect of BCAA catabolic enzyme expression at the mRNA or protein level. Similarly, despite reducing mitochondrial metabolism, rapamycin also had no significant effect on mRNA or protein expression of BCAA catabolic enzymes. Furthermore, rapamycin did not alter relative activity (phosphorylation status) of BCKDHa, which we originally expected given previous findings from rodents treated with rapamycin [42]. That said, clear and important differences exist between the fasting mice model used by Zhen et al. and the in vitro model employed by the current report [42]. Finally, we observed no effect of either treatment condition on extracellular BCAA content, suggesting that BCAA uptake and use were similar between groups. 

Collectively, mTOR signaling is highly influential in the regulation of cell metabolism and, as such, has emerged as a prominent molecular target in preventing and treating metabolic disease. BCAAs are molecules that appear to stimulate mTOR activity and whose metabolism may be governed by mTOR. Our report demonstrates that inhibition of mTOR via rapamycin suppresses cell metabolism but that treatment of cells with the reported mTOR agonist MHY1485 did not enhance or restore metabolism in control or rapamycin-treat cells, respectively. Furthermore, we did not observe any effect of mTOR manipulation on indicators of BCAA metabolism. This data may suggest that enhanced BCAA catabolic enzyme expression by leucine may be a function of leucine stimulating mTOR and its own metabolism through different mechanisms [42]. That said, Zhen et al., did demonstrate that rapamycin reduced BCKDH activity following rapamycin treatment, suggesting that mTOR may in part regulate BCAA metabolism under select experimental conditions [42]. 

## 4. Materials and Methods

### 4.1. Cell Culture

C2C12 mouse myoblasts from ATCC (Manassas, VA, USA) were cultured in Dulbecco’s Modified Eagle’s Medium (DMEM) containing 4500 mg/L glucose and 20% heat-inactivated fetal bovine serum (FBS) and 100 U/mL penicillin and 100 µg/mL streptomycin in a humidified 5% CO_2_ atmosphere at 37 °C. Cells were grown to confluency with growth media changed every two to three days (using cell passages < 20 for all experiments). Differentiation was accomplished by replacing growth media with DMEM with 2% horse serum and 100 U/mL penicillin and 100 µg/mL streptomycin for 6–9 days [47]. MHY1485 from Cayman Chemical was dissolved in 100% DMSO, and cells were treated with a final concentration of 10μM for MHY1485 based on previous observations demonstrating effective mTORC activation in C2C12 cells [45]. To identify if any responses were dependent on mTORC activation, rapamycin from Cayman Chemical was dissolved in DMSO, and cells were then treated with either DMSO or MHY1485 at 10μM either with DMSO or rapamycin (RAPA) at 100 nM (with final DMSO at 0.2% *vol*:*vol* for all cells) for 24 h. For insulin-sensitivity Western blot experiments, cells were treated as described above and stimulated with insulin at 100 nM for 30 min in serum-free media or negative control (serum-free media only). 

### 4.2. Myotube Fusion Index

Following treatment as described above, cells were fixed in media containing 3.7% formaldehyde at 37 °C for 60 min. Cells were then stained with DAPI at 0.5 µM in PBS for 10 min in the dark and fluorescence quantified (360/460 nm), as shown in Figure S1. Myotubes were then permeabilized with PBS containing 0.1% Triton 100X for 5 min, followed by staining with phalloidin-FITC conjugate from Cayman Chemical (0.1% in PBS) for approximately 30 min as previously performed [48]. Cells were then thoroughly rinsed and imaged using the 40× objective. The myotube fusion index was calculated as previously described as the percentage of nuclei integrated into myotubes [49] (defined as visible myotubes containing 3 or more nuclei). Counting of total nuclei was performed by two blinded members of the research team. 

### 4.3. Quantitative Real Time Polymerase Chain Reaction (qRT-PCR)

Following treatment, total mRNA was extracted using the Trizol method and quantified (via NanoDrop from Thermo Fisher, Wilmington, DE, USA), and cDNA was synthesized using the iScript cDNA Synthesis Kit from Bio-Rad (Hercules, CA, USA) according to the manufacturer’s instructions. PCR primers were synthesized by Integrated DNA Technologies (Coralville, IA, USA) (Table S1). Amplification of target genes were normalized to the housekeeping gene, TATA-binding protein (*Tbp*), which did not differ between groups (Figure S2). qRT-PCR reactions were performed using the CFX Connect System from Bio-Rad (Hercules, CA). SYBR Green-based PCR was performed using final primer concentrations at 3.75 µM in a total volume of 10 µL per well. The following cycling parameters were used: 95 °C for 3 min followed by 40 cycles of 95 °C for 15 s and 60 °C for 30 s. qRT-PCR reactions were performed using n = 3 per treatment condition from 2 independent experiments with n = 6 for the final analysis. Relative quantification was determined via the ^ΔΔ^Ct method, as previously performed [47]. 

### 4.4. Immunoblotting 

Following treatment as described above, whole cell lysates were then prepared by harvesting the cells on ice in RIPA buffer supplemented with protease inhibitor, followed by incubation on ice for 60 min. Insoluble material was removed, and protein concentrations were determined by Bradford assay. Total protein (50 μg per sample) was size-separated by 10% sodium dodecyl sulfate polyacrylamide gel electrophoresis (SDS-PAGE) and electro-transferred to PVDF membranes. After blocking in TBST-5% non-fat milk powder for 1 h, membranes were probed at 4 °C overnight with primary antibodies in TBST-5% non-fat milk powder (details in Table S2). Bound antibodies were detected by horseradish peroxidase-conjugated secondary antibodies from AbCam (Cambridge, MA, USA) at a dilution of 1:5000 in TBST-5% non-fat milk powder for 1 h at room temperature while shaking. Protein signal intensities were determined by chemiluminescence using the Clarity Western ECL substrate kit from Bio-Rad (Hercules, CA, USA) and imaged using the ChemiDoc Touch from Bio-Rad (Hercules, CA, USA). Relative signal intensities were quantified using Image Lab from Bio-Rad (Hercules, CA, USA), as previously performed [47]. Blots were performed using 3 replicates per condition performed across 2 independent experiments with n = 6 for the final analysis. Molecular weights for all targets were verified against sizes suggested by product brochures. Importantly, loading of targets did not differ between groups (Figure S3). 

### 4.5. Seahorse Metabolic Assays

Cells were seeded into Seahorse XFe96 culture plates, differentiated, and treated as described above. Media were then replaced with XF Assay Media obtained from Agilent Technologies (Santa Clara, CA, USA) containing glucose at 25 mM, pyruvate at 1mM, and glutamine at 2 mM. Following incubation, baseline measurements of oxygen consumption rate (OCR) and extracellular acidification rate (ECAR) were recorded as indicators of basal oxidative metabolism and glycolytic metabolism, respectively. Following basal measurements, each well was infused with oligomycin (an inhibitor of ATP synthase) at a final concentration of 2 μM to induce maximal glycolytic metabolism. Cells were then exposed to carbonyl cyanide p-[trifluoromethoxy]-phenyl-hydrazone (FCCP) at 2 μM to uncouple electron transport and induce peak OCR. Maximal respiration measurements were followed by the injection of rotenone at 1 μM to reveal non-mitochondrial respiration. The Seahorse XFe96 Analyzer was run using a 6 min cyclic protocol command (mix for 3 min and measure for 3 min). MitoStress assays included n = 23 per group repeated with 2 independent experiments for n = 46 per group for the final analysis. States of mitochondrial metabolism were calculated by subtracting non-mitochondrial respiration from basal or FCCP-induced peak mitochondrial oxygen consumption, as previously performed [47]. No wells showed negative OCR values or lack of response to injection.

### 4.6. Fluorescent Staining and Microscopy

Immediately following the Seahorse metabolic assay described above, cells were fixed using 3.7% formaldehyde at 37 °C with a 5% CO_2_ atmosphere. The fixing agent was then removed, and cells were stained with DAPI at 0.5 µM in dH_2_O, and fluorescence was measured at 360/460 nM (Figure S4). Cells were then stained with 100 µM nonyl acridine orange (NAO) (Fremont, CA, USA) in dH_2_O and incubated in the dark at room temperature for 10 min. Fluorescence was then measured using 485/525 nm excitation/emission. Neutral lipid content was measured using Nile Red staining at 10 µM dH_2_O with 1% DMSO vol/vol using 530/645 nm excitation/emission and did not differ between groups (Figure S5). All fluorescent measurements were made in triplicate and the average (less background) analyzed with n = 23 per group repeated with 2 independent experiments with n = 46 per group for the final analyses. Following fluorescent quantification, cells were imaged using the 10× and/or 20× objective using the Motic AE31E inverted microscope and Moticam Pro 252B (Causeway Bay, Hong Kong, China), as previously performed [47]. 

### 4.7. Liquid Chromatography–Mass Spectrometry (LC–MS)

As previously performed [47], chromatographic separation and quantification of leucine, isoleucine, and valine was performed using a Shimadzu Nexera UHPLC system equipped with a Phenomenex Kinetex C18 100 Å column (100 × 3mm, 2.6 µm) kept at a temperature of 30 °C connected to a Shimadzu LCMS-8045 triple quadrupole mass spectrometer (Shimadzu, Kyoto, Japan) fitted with a DUIS ion source [50]. The source used nebulizer gas 2.0 L/minute, drying gas 10.0 L/minute, desolvation line (DL) temperature 250 °C, and heat block temperature 400 °C, with CID gas 230 kPa. The mobile phases of A (water with 0.1% formic acid) and B (methanol 0.1% formic acid) were used at a flow rate of 0.4 mL/min for the following gradient method: 0 min, 20% B; 1.7 min, 40% B; 5.0 min, 65% B; 8.0 min, 65% B; followed by 4 min 20% B for column equilibration. The injection volume was maintained at 1 µL. This afforded reproducible retention time values for valine (1.589 min), isoleucine (2.093 min), and leucine (2.213 min).

Shimadzu LabSolution software version 5.97 was used to acquire and process the data. The fragmentation for each BCAA was optimized using MRM set to positive mode for valine (118.1 to 72.2 *m*/*z*, Q1 −23.0 V, CE −12.0 V, and Q3 −20.0 V), isoleucine (132.0 to 69.2 *m*/*z*, Q1 −10.0 V, CE −19.0 V, and Q3 −11.0 V), and leucine (132.1 to 43.2 *m*/*z*, Q1 −10.0 V, CE −26.0 V, and Q3 −18.0 V), with a dwell time of 100 msec.

A stock solution containing all BCAAs at a concentration of 8.0 mM was obtained by dissolving each amino acid in water/methanol solution (50:50, *v*/*v*) and kept at 4 °C. Further dilutions with water/methanol were performed to assemble a calibration curve ranging from 3.125 to 100.0 µM. Experiments were performed using 3 replicates per group for each of 2 independent cell culture experiments with n = 6 for each group in the final analyses.

### 4.8. Statistical Analyses

Data are presented as dot plots with group means or as group means ± SE. Data were analyzed with two-way ANOVA with subsequent one-way ANOVA with Bonferroni’s correction for pairwise group differences. For small data sets (n < 24), data were analyzed using Prism 3.0. Larger data sets were analyzed using SPSS Version 29.0.0.0. Values of *p* < 0.05 were used to identify significant differences between groups. For clarity of sample size calculations, independent experiments indicate different versions of cells seeded and treated independently of other experiments, while independent biological sample/replicates are represented by independent wells within each experiment. Technical replicates represent multiple measurements within a single sample or well, and the averages of all technical replicates for each biological sample were used as n = 1.

## 5. Conclusions

Collectively, our report verified that inhibition of mTOR via rapamycin suppresses cell metabolism; however, we did not observe any effect of mTOR inhibition on BCAA metabolism. This may indicate that previous observations showing enhanced BCAA catabolic enzyme activity by leucine may be a consequence of leucine stimulating its own metabolism. Further research will be necessary to assess this speculation as well as to determine if the ability of MHY1485 to stimulate mTOR activity varies between cell/tissue types.

## Data Availability

The data that support the findings of this study are presented within the manuscript and Supplemental Materials, and additional information is available from the corresponding author upon reasonable request.

## Supplementary Materials

The supporting information can be downloaded at https://www.mdpi.com/article/10.3390/ijms25136819/s1.
