# Supplementary material for: Effect of mTORC Agonism via MHY1485 with and without Rapamycin on C2C12 Myotube Metabolism
Source: Int J Mol Sci. 2024 Jun 21;25(13):6819. doi: 10.3390/ijms25136819 (PMC11241331; doi:10.3390/ijms25136819)
Supplement: Supplementary file 1 [file ijms-25-06819-s001.zip › ijms-3045254-supplementary.pdf]

Supplemental Materials

Effect of mTORC Agonism via MHY1485 with and without Rapamycin on C2C12 Myotube Metabolism

Norah E. Cook [1], Macey R. McGovern [1], Toheed Zaman [2], Pamela M. Lundin [2] and Roger A. Vaughan [1,*]

[1] Department of Health and Human Performance, High Point University, High Point, NC 27262-3598, USA; ncook@highpoint.edu (N.E.C.); mmcgove4@highpoint.edu (M.R.M.)
[2] Department of Chemistry, High Point University, High Point, NC 27262-3598, USA; tzaman@highpoint.edu (T.Z.); plundin@highpoint.edu (P.M.L.)
* Correspondence: rvaughan@highpoint.edu; Tel.: +1-336-841-9688

Supplemental Figures

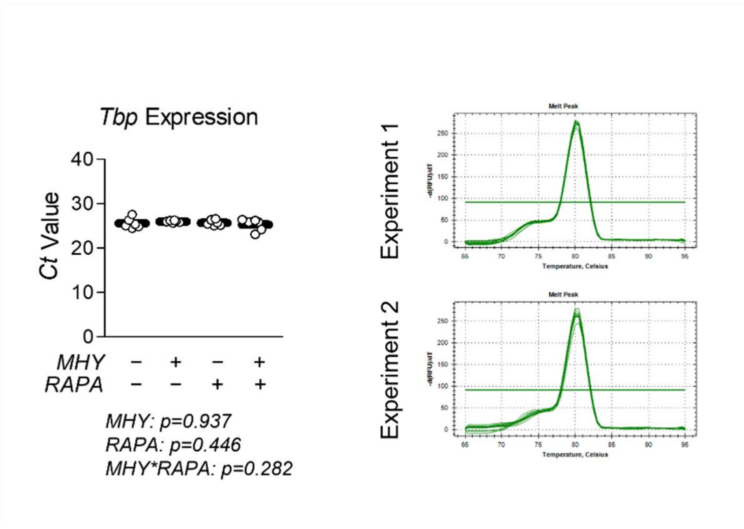

Fig s1. Effect of MHY1485 on myotube mRNA expression of *Tbp* (qRT-PCR loading control). Effect of MHY1485 (MHY) at 10μM both with and without rapamycin (RAPA) at 100nM (final concentration of DMSO at 0.2% for all samples) for 24 hours on Tata binding protein (*Tbp*) expression which did not differ between groups.

NOTES: Data were analyzed using two-way ANOVA followed by one-way ANOVA with Bonferroni's correction for multiple comparisons to assess differences in each *Tbp* expression. Data were generated from 3 replicates per group across 2 independent experiments with n=6 for the final analysis.

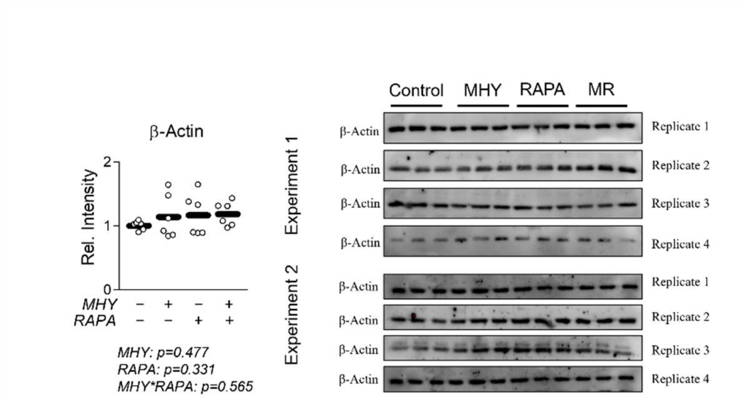

Fig s2. Effect of MHY1485 on myotube β-Actin expression (Western blot loading control). Effect of MHY1485 (MHY) at 10μM both with and without rapamycin (RAPA) at 100nM (final concentration of DMSO at 0.2% for all samples) for 24 hours on β-Actin expression which did not differ between groups.

NOTES: Data were analyzed using two-way ANOVA followed by one-way ANOVA with Bonferroni's correction for multiple comparisons to assess differences in each β-Actin expression. Data were generated from 3 replicates per group across 2 independent experiments with n=6 for the final analysis.

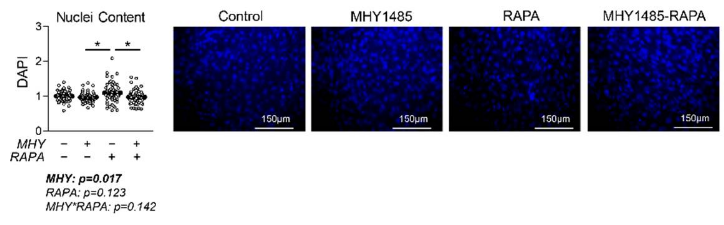

Fig s3. Effect of MHY1485 on myotube nuclei content during Seahorse assay. Effect of MHY1485 (MHY) at 10µM both with and without rapamycin (RAPA) at 100nM (final concentration of DMSO at 0.2% for all samples) for 24 hours on myotube nuclei content following the Seahorse metabolic assay.
NOTES: Data were analyzed using two-way ANOVA followed by one-way ANOVA with Bonferroni's correction for multiple comparisons to assess differences in nuclei content. * indicates a significant difference between groups upon pair-wise comparisons. Data were generated from 23 replicates per group across 2 independent experiments with n=46 for the final analysis.

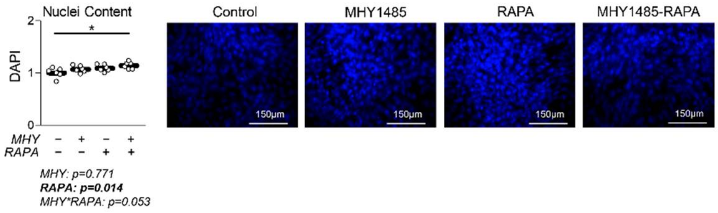

Fig s4. Effect of MHY1485 on myotube nuclei content for myotube fusion index and media BCAA content. Effect of MHY1485 (MHY) at 10µM both with and without rapamycin (RAPA) at 100nM (final concentration of DMSO at 0.2% for all samples) for 24 hours on myotube nuclei content.
NOTES: Data were analyzed using two-way ANOVA followed by one-way ANOVA with Bonferroni's correction for multiple comparisons to assess differences in nuclei content. * indicates a significant difference between groups upon pair-wise comparisons. Data were generated from 3 replicates per group across 2 independent experiments with n=6 for the final analysis.

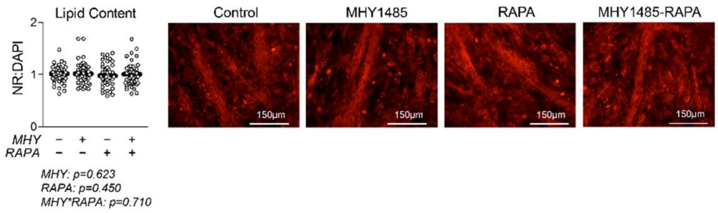

Fig s5. Effect of MHY1485 on myotube lipid content. Effect of MHY1485 (MHY) at 10µM both with and without rapamycin (RAPA) at 100nM (final concentration of DMSO at 0.2% for all samples) for 24 hours on myotube lipid content indicated by Nile red (NR) staining.
NOTES: Data were analyzed using two-way ANOVA followed by one-way ANOVA with Bonferroni's correction for multiple comparisons to assess differences in lipid content. Data were generated from 23 replicates per group across 2 independent experiments with n=46 for the final analysis.

Supplemental Tables

Table s1 Summary of qRT-PCR primers from Integrated DNA Technologies (Coralville, IA). Abbreviations: branched-chain aminotransferase 2 (*Bcat2*), branched-chain alpha-keto acid dehydrogenase (*Bckdha*), citrate synthase (*Cs*), glucose transporter 4 (*Slc2a4* or *Glut4*), lactate dehydrogenase a (*Ldha*), lactate dehydrogenase b (*Ldhb*), myosin heavy chain 7 (MYH7), nuclear respiratory factor 1 (*Nrf1*), pyruvate dehydrogenase (*Pdh*), peroxisome proliferator-activated receptor gamma coactivator 1 alpha (*Ppargc1a),* TATA box binding protein (*Tbp*), and mitochondrial transcription factor A (*Tfam*).

Gene Abbreviation	*Forward Sequence*	*Reverse Sequence*
Bcat2	5'-CGGACCCTTCATTCGTCAGA-3'	5'-CCATAGTTCCCCCCCAACTT-3'
Bckdha	5'-CCAGGGTTGGTGGGATGAG-3'	5'-GGCTTCCATGACCTTCTTTCG-3'
Cs	5'-TGAGAGGCATGAAGGGACTTGTGT-3'	5'-ATCTGTCCAGTTACCAGCAGCCAA-3'
Slc2a4 (Glut4)	5'-GATGAGAAACGGAAGTTGGAGAGA-3'	5'-GCACCACTGCGATGATCAGA-3'
Ldha	5′-GGCTTGTGCCATCAGTATCT-3'	5′-CCCGCCTAAGGTTCTTCATTAT-3′
Ldhb	5'-AGTCTCCCGTGCATCCTCAA-3'	5'-AGGGTGTCCGCACTCTTCCT-3'
Myh7	5'-CAAGCAGCAGTTGGATGAGCGACT -3'	5'-TCCTCCAGCTCCTCGATGCGT-3'
Nrf1	5'-ACCCTCAGTCTCACGACTAT-3'	5'-GAACACTCCTCAGACCCTTAAC-3'
Pdh	5'-GAAGGCCCTGCATTCAACTTC-3'	5'-ATAGGGACATCAGCACCAGTGA-3'
Ppargc1a	5'-GACAATCCCGAAGACACTACAG-3'	5'-AGAGAGGAGAGAGAGAGAGAGA-3'
Tbp	5'-GGGATTCAGGAAGACCACATA-3'	5'-CCTCACCAACTGTACCATCAG-3'
Tfam	5'-GAAGGGAATGGGAAAGGTAGAG-3'	5'-ACAGGACATGGAAAGCAGATTA-3'

Table s2 Summary of primary antibodies used for western blot experiments. Abbreviations: branched-chain aminotransferase 2 (BCAT2), branched-chain alpha-keto acid dehydrogenase E1α (BCKDHE1α), citrate synthase (CS), mouse monoclonal (MM), mechanistic target of rapamycin (mTOR), myosin heavy chain 3 (MYH3) nuclear respiratory factor 1 (NRF1), peroxisome proliferator-activated receptor gamma coactivator 1 alpha (PGC-1α), rabbit polyclonal (RP), and mitochondrial transcription factor A (TFAM). *Notes: Target molecular weight was based on product datasheet. Molecular weights for all targets were verified against sizes suggested by product brochures.*

Protein Target	*Type*	*Dilution*	*Company*	*Item*	*Approx. Mol Wt.*	*Product Link*
pAkt (Ser 473)	RP	1:1000	SC Biotechnology	sc-7985-R	62kd	p-Akt1/2/3 (Ser 473)
Akt	RP	1:1000	Cell Signaling	9272	62kd	Akt Antibody#9272
β-Actin	RP	1:1000	SC Biotechnology	sc-130656	43kd	Datasheet
BCAT2	RP	1:1000	Bioss	BS-6589R	44kd	Datasheet
pBCKDHa (Ser 293)	RP	1:1000	AbCam	ab200577	50kd	Phospho BCKDHA (S293)
CS	MM	1:1000	SC Biotechnology	sc-390693	52kd	sc-390693
MYH3	MM	1:500	SC Biotechnology	sc-376157	200kd	Datasheets
pmTOR (Ser 2448)	RP	1:1000	SC Biotechnology	sc-101738	220kd	p-mTOR (Ser 2448)
NRF1	RP	1:1000	SC Biotechnology	sc-33771	68kd	Datasheet
PGC-1α	RP	1:1000	SC Biotechnology	sc-13067	90kd	PGC-1α (H-300)
TFAM	RP	1:1000	SC Biotechnology	sc-28200	25kd	Datasheet
